# *N*-(4-Meth­oxy-2-nitro­phen­yl)acetamide

**DOI:** 10.1107/S2414314622002772

**Published:** 2022-03-17

**Authors:** James E. Hines III, Curtistine J. Deere, Poornasai Vaddi, Ranjeeth R. Kondati, Frank R. Fronczek, Rao M. Uppu

**Affiliations:** a Department of Environmental Toxicology, Southern University and A&M College, Baton Rouge, LA 70813, USA; b Department of Chemistry, Louisiana State University, Baton Rouge, LA 70803, USA; University of Aberdeen, Scotland

**Keywords:** crystal structure, hydrogen bonding

## Abstract

In the title compound, C_9_H_10_N_2_O_4_, the three substituents vary in the degree of lack of planarity with the central phenyl ring. The meth­oxy group is nearest to being coplanar, with a C—C—O—C torsion angle of 6.1 (5)°. The nitro group is less coplanar, with a 12.8 (5)° twist about the C—N bond and the acetamido group is considerably less coplanar with the central ring, having a 25.4 (5)° twist about the C—N bond to the ring. The NH group forms an intra­molecular N—H⋯O hydrogen bond to a nitro-group O atom.

## Abstract 2

In the title compound, the pendant groups differ in their divergence from coplanarity with the central ring.

## Graphical abstract

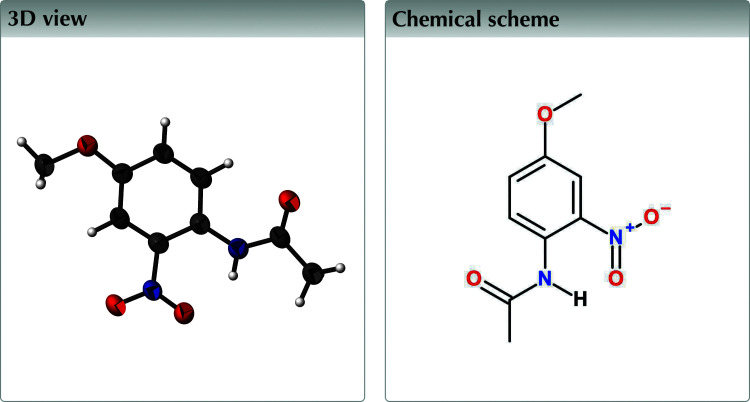

## Structure description

The analgesic use of 4-alk­oxy­acetanilides, in particular 4-eth­oxy­acetanilide or 4-EA, predates the First World War. 4-Hy­droxy­acetanilide (popularly known as Tylenol or acetamino­phen) and 4-EA were introduced into the markets at around the same time; however, 4-EA was withdrawn from sale some decades ago due to its carcinogenic and kidney-damaging properties (Dubach *et al.*, 1983 ▸; Nakanishi *et al.*, 1982 ▸). Although there has been extensive information on phase I and phase II biotransformation of 4-alk­oxy­acetanilides (Hinson, 1983 ▸; Kapetanović *et al.*, 1979 ▸; Mulder *et al.*, 1984 ▸; Veronese *et al.*, 1985 ▸), little or no information is available on nitrated or other oxidation products that could be formed in reactions with cellular oxidants, such as hypochlorite (^−^OCl)/hypo­chlorous acid (HOCl; p*K*_a_ ≃ 7.53) and per­oxy­nitrite (ONOO^−^)/per­oxy­nitrous acid (ONOOH; p*K*_a_ ≃ 6.2; ONOOH and ONOO^−^ are collectively referred to as per­oxy­nitrite or PN). We have shown, for instance, that 4-hy­droxy­acetanilide forms nitrated and chlorinated products along with varying amounts of dimers when reacted with HOCl/^−^OCl and PN/CO_2_ under physiologically relevant conditions (Uppu & Martin, 2005 ▸; Deere *et al.*, 2022 ▸). We suspect that similar products (or their positional isomers) may be formed in the reactions of 4-alk­oxy­acetanilides with the cellular oxidants referenced above. Towards a better understanding of this and to shed light on mol­ecular targets (Bertolini *et al.*, 2006 ▸), we have synthesized the title compound, C_9_H_10_N_2_O_4_: single crystals grown from aqueous solution were analyzed by X-ray diffraction.

**Figure 1:**
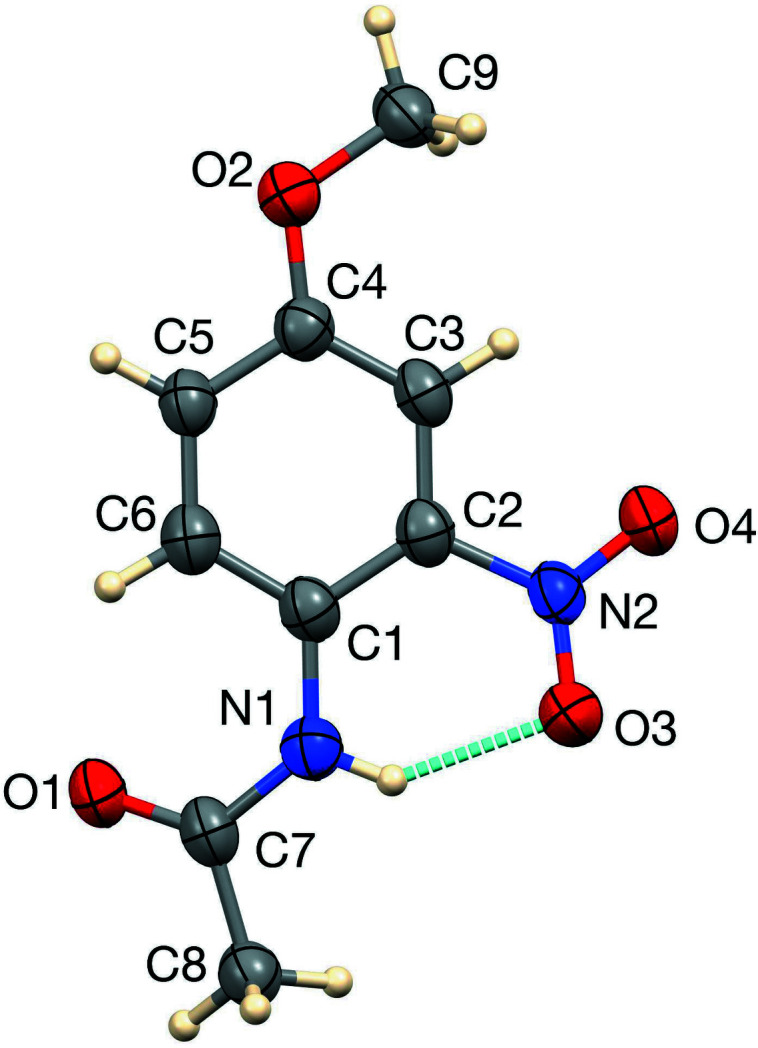
The title mol­ecule with 50% displacement ellipsoids with the intra­molecular N—H⋯O hydrogen bond shown as a blue dashed line.

The title compound is shown in Fig. 1 ▸. It is significantly non-planar, and its deviation from planarity may be described by torsion angles about bonds from the central C1–C6 phenyl ring to the three substituents. The meth­oxy group is nearest to being coplanar, with a C9—O2—C4–C3 torsion angle of 6.1 (5)°. The nitro group deviates more from coplanarity with the central ring, with the O3—N2—C2—C1 torsion angle being −12.8 (5)°. The acetamido group is considerably less coplanar with the central ring, with a C7—N1—C1—C6 torsion angle of 25.4 (5)°. These deviations are similar to those seen in the analogous 4-eth­oxy compound (Uppu *et al.*, 2020 ▸), in which the corresponding torsion angles are 0.56 (12), −14.94 (13) and 18.23 (15)°, respectively. *N*-(4-Hy­droxy-2-nitro­phen­yl)acetamide (Hines *et al.*, 2022 ▸) is considerably more planar, with torsion angles to the nitro group and to the acetamido group being −0.79 (19) and 3.1 (2)°, respectively, likely as a result of inter­molecular hydrogen bonding by the OH group. The structure of *N*-(4-hy­droxy-3-nitro­phen­yl)acetamide, in which the OH group likewise participates in inter­molecular hydrogen bonding, has also been reported (Salahifar *et al.*, 2015 ▸; Deere *et al.*, 2019 ▸). It is also more planar than the title compound, with a torsion angle of −11.8 (2)° for the nitro group and 9.0 (2)° for the acetamido group. An intra­molecular N1—H1*N*⋯O3 hydrogen bond (Table 1 ▸) is observed in the title compound.

**Figure 2:**
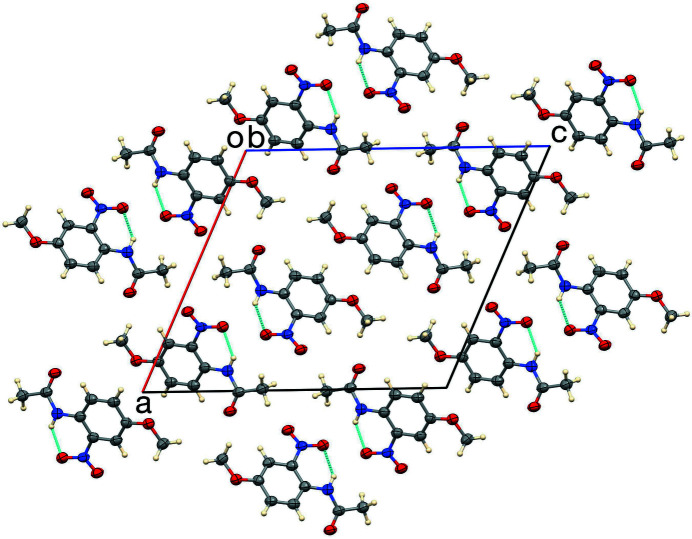
The unit cell, viewed down the [010] direction, showing intra­molecular hydrogen bonds.

The unit cell of the title compound is shown in Figs. 2 ▸ and 3 ▸. The closest inter­molecular contact is C5—H5⋯O2 (at 1 − *x*, −*y*, 1 − *z*), forming dimers about inversion centers with a C⋯O distance of 3.418 (4) Å and 171° angle about H. Mol­ecules form a herringbone pattern in the [101] direction with alternate phenyl rings forming a dihedral angle of 65.7 (2)°.

## Synthesis and crystallization

*N*-(4-Meth­oxy-2-nitro­phen­yl)acetamide was synthesized by acetyl­ation of 4-meth­oxy-2-nitro­aniline using acetic anhydride in acetic acid solvent: 3.36 g (20 mmol) of 4-meth­oxy-2-nitro­aniline in 30 ml of glacial acetic was allowed to react with 2.46 g (24 mmol) of acetic anhydride for 18 h at room temperature. The reaction mixture was stirred continuously during the reaction. In the end, the mixture was dried under vacuum, and the *N*-(4-meth­oxy-2-nitro­phen­yl)acetamide in the residue was purified by recrystallization twice from aqueous solution. Single crystals in the form of yellow laths were grown in water by slow cooling of a hot and nearly saturated solution of the title compound.

## Refinement

Crystal data, data collection and structure refinement details are summarized in Table 2 ▸.

## Supplementary Material

Crystal structure: contains datablock(s) I. DOI: 10.1107/S2414314622002772/hb4403sup1.cif

Structure factors: contains datablock(s) I. DOI: 10.1107/S2414314622002772/hb4403Isup2.hkl

Supporting information file. DOI: 10.1107/S2414314622002772/hb4403Isup3.cml

CCDC reference: 2157748

Additional supporting information:  crystallographic information; 3D view; checkCIF report

## Figures and Tables

**Figure 3:**
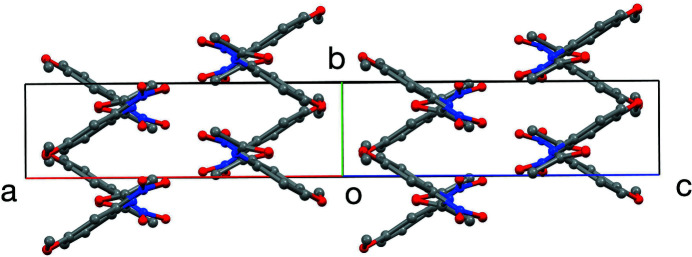
The unit cell, viewed down the [101] direction. H atoms are not shown.

**Table 1:** Hydrogen-bond geometry (Å, °)

*D*—H⋯*A*	*D*—H	H⋯*A*	*D*⋯*A*	*D*—H⋯*A*
N1—H1*N*⋯O3	0.87 (5)	1.92 (5)	2.632 (4)	137 (4)
C5—H5⋯O2^i^	0.95	2.48	3.418 (4)	171
C6—H6⋯O1	0.95	2.30	2.864 (4)	117
C8—H8*B*⋯O3^ii^	0.98	2.64	3.578 (4)	160
C8—H8*C*⋯O4^iii^	0.98	2.63	3.546 (4)	156

Symmetry codes: (i) 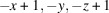 ; (ii) 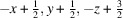 ; (iii) 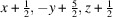 .

**Table 2:** Experimental details

Crystal data	
Chemical formula	C_9_H_10_N_2_O_4_
*M*_r_	210.19
Crystal system, space group	Monoclinic, *P*2_1_/*n*
Temperature (K)	90
*a*, *b*, *c* (Å)	14.8713 (7), 3.9563 (2), 17.2057 (9)
β (°)	114.051 (3)
*V* (Å^3^)	924.42 (8)
*Z*	4
Radiation type	Cu *K*α
μ (mm^−1^)	1.03
Crystal size (mm)	0.42 × 0.06 × 0.01

Data collection	
Diffractometer	Bruker Kappa APEXII DUO CCD
Absorption correction	Multi-scan (*SADABS*; Krause *et al.*, 2015 ▸)
*T*_min_, *T*_max_	0.692, 0.990
No. of measured, independent and observed [*I* > 2σ(*I*)] reflections	11516, 1638, 1211
*R*_int_	0.122
(sin θ/λ)_max_ (Å^−1^)	0.595

Refinement	
*R*[*F* ^2^ > 2σ(*F* ^2^)], *wR*(*F* ^2^), *S*	0.071, 0.203, 1.09
No. of reflections	1638
No. of parameters	141
H-atom treatment	H atoms treated by a mixture of independent and constrained refinement
Δρ_max_, Δρ_min_ (e Å^−3^)	0.24, −0.27

Computer programs: *APEX2* and *SAINT* (Bruker, 2016 ▸), *SHELXT2014/5* (Sheldrick, 2008 ▸), *SHELXL2017/1* (Sheldrick, 2015 ▸), *Mercury* (Macrae *et al.*, 2020 ▸), and *publCIF* (Westrip, 2010 ▸).

## full crystallographic data

### Crystal data

**Table:**

C_9_H_10_N_2_O_4_	*F*(000) = 440
*M**_r_* = 210.19	*D*_x_ = 1.510 Mg m^−^^3^
Monoclinic, *P*2_1_/*n*	Cu *K*α radiation, λ = 1.54184 Å
*a* = 14.8713 (7) Å	Cell parameters from 2102 reflections
*b* = 3.9563 (2) Å	θ = 3.3–66.3°
*c* = 17.2057 (9) Å	µ = 1.03 mm^−^^1^
β = 114.051 (3)°	*T* = 90 K
*V* = 924.42 (8) Å^3^	Lath, yellow
*Z* = 4	0.42 × 0.06 × 0.01 mm

### Data collection

**Table:**

Bruker Kappa APEXII DUO CCD diffractometer	1638 independent reflections
Radiation source: IµS microfocus	1211 reflections with *I* > 2σ(*I*)
QUAZAR multilayer optics monochromator	*R*_int_ = 0.122
φ and ω scans	θ_max_ = 66.7°, θ_min_ = 3.3°
Absorption correction: multi-scan (SADABS; Krause *et al.*, 2015)	*h* = −17→17
*T*_min_ = 0.692, *T*_max_ = 0.990	*k* = −4→4
11516 measured reflections	*l* = −20→20

### Refinement

**Table:**

Refinement on *F*^2^	0 restraints
Least-squares matrix: full	Hydrogen site location: mixed
*R*[*F*^2^ > 2σ(*F*^2^)] = 0.071	H atoms treated by a mixture of independent and constrained refinement
*wR*(*F*^2^) = 0.203	*w* = 1/[σ^2^(*F*_o_^2^) + 0.298*P*] where *P* = (*F*_o_^2^ + 2*F*_c_^2^)/3
*S* = 1.09	(Δ/σ)_max_ < 0.001
1638 reflections	Δρ_max_ = 0.24 e Å^−^^3^
141 parameters	Δρ_min_ = −0.27 e Å^−^^3^

### Special details

**Table:**

Geometry. All esds (except the esd in the dihedral angle between two l.s. planes) are estimated using the full covariance matrix. The cell esds are taken into account individually in the estimation of esds in distances, angles and torsion angles; correlations between esds in cell parameters are only used when they are defined by crystal symmetry. An approximate (isotropic) treatment of cell esds is used for estimating esds involving l.s. planes.
Refinement. All H atoms were located in difference maps and those on C were thereafter treated as riding in geometrically idealized positions with C—H distances 0.95 Å for phenyl and 0.98 Å for methyl. Coordinates of the N—H hydrogen atom were refined. *U*_iso_(H) values were assigned as 1.2*U*_eq_ for the attached atom (1.5 for methyl).

### Fractional atomic coordinates and isotropic or equivalent isotropic displacement parameters (Å^2^)

**Table:**

	*x*	*y*	*z*	*U*_iso_*/*U*_eq_	
O1	0.58485 (18)	0.7604 (7)	0.81731 (16)	0.0512 (7)	
O2	0.36866 (18)	0.2368 (6)	0.43490 (15)	0.0455 (6)	
O3	0.23210 (18)	0.9412 (7)	0.67288 (16)	0.0529 (7)	
O4	0.16683 (18)	0.9595 (6)	0.53559 (16)	0.0480 (7)	
N1	0.4165 (2)	0.7462 (7)	0.75182 (18)	0.0447 (7)	
H1N	0.363 (4)	0.830 (11)	0.753 (3)	0.054*	
N2	0.2326 (2)	0.8718 (7)	0.60286 (18)	0.0416 (7)	
C1	0.4018 (3)	0.6185 (9)	0.6716 (2)	0.0428 (8)	
C2	0.3144 (2)	0.6751 (8)	0.5992 (2)	0.0420 (8)	
C3	0.3005 (2)	0.5555 (8)	0.5182 (2)	0.0415 (8)	
H3A	0.241073	0.602472	0.470197	0.050*	
C4	0.3736 (3)	0.3699 (8)	0.5092 (2)	0.0417 (8)	
C5	0.4605 (3)	0.3057 (8)	0.5806 (2)	0.0431 (8)	
H5	0.511084	0.176401	0.574482	0.052*	
C6	0.4740 (3)	0.4264 (9)	0.6593 (2)	0.0442 (8)	
H6	0.533915	0.378381	0.706654	0.053*	
C7	0.5054 (3)	0.8178 (8)	0.8187 (2)	0.0435 (8)	
C8	0.4928 (3)	0.9743 (9)	0.8926 (2)	0.0475 (8)	
H8A	0.473964	0.799894	0.923540	0.071*	
H8B	0.441299	1.147414	0.872021	0.071*	
H8C	0.554993	1.078496	0.930706	0.071*	
C9	0.2843 (3)	0.3245 (9)	0.3589 (2)	0.0488 (9)	
H9A	0.224837	0.228708	0.361637	0.073*	
H9B	0.292327	0.233462	0.309172	0.073*	
H9C	0.278101	0.571021	0.354165	0.073*	

### Atomic displacement parameters (Å^2^)

**Table:**

	*U*^11^	*U*^22^	*U*^33^	*U*^12^	*U*^13^	*U*^23^
O1	0.0331 (14)	0.0634 (15)	0.0497 (14)	0.0015 (10)	0.0093 (11)	−0.0066 (11)
O2	0.0413 (14)	0.0494 (13)	0.0439 (13)	−0.0005 (10)	0.0154 (10)	−0.0013 (10)
O3	0.0419 (14)	0.0706 (16)	0.0434 (14)	0.0026 (12)	0.0145 (11)	−0.0053 (11)
O4	0.0361 (13)	0.0553 (14)	0.0444 (13)	0.0047 (10)	0.0079 (10)	0.0026 (10)
N1	0.0398 (17)	0.0513 (16)	0.0424 (15)	0.0000 (12)	0.0162 (13)	0.0006 (12)
N2	0.0308 (15)	0.0463 (15)	0.0425 (16)	−0.0030 (11)	0.0097 (13)	−0.0028 (12)
C1	0.0376 (18)	0.0453 (17)	0.0429 (18)	−0.0019 (13)	0.0140 (15)	0.0030 (13)
C2	0.0354 (18)	0.0430 (17)	0.0458 (19)	−0.0024 (13)	0.0148 (15)	0.0032 (13)
C3	0.0342 (17)	0.0411 (17)	0.0432 (17)	−0.0037 (13)	0.0095 (14)	0.0033 (13)
C4	0.0381 (18)	0.0425 (17)	0.0446 (18)	−0.0025 (13)	0.0170 (15)	−0.0011 (13)
C5	0.0331 (18)	0.0454 (17)	0.0469 (19)	−0.0008 (13)	0.0125 (15)	−0.0009 (14)
C6	0.0366 (18)	0.0437 (17)	0.0495 (19)	0.0007 (13)	0.0146 (15)	0.0033 (14)
C7	0.0342 (19)	0.0449 (17)	0.0452 (18)	−0.0005 (13)	0.0099 (15)	0.0039 (13)
C8	0.0412 (19)	0.0501 (19)	0.0462 (18)	0.0005 (15)	0.0126 (15)	−0.0017 (15)
C9	0.045 (2)	0.0517 (19)	0.0416 (18)	0.0030 (15)	0.0089 (16)	−0.0008 (14)

### Geometric parameters (Å, º)

**Table:**

O1—C7	1.214 (4)	C3—H3A	0.9500
O2—C4	1.357 (4)	C4—C5	1.395 (5)
O2—C9	1.438 (4)	C5—C6	1.371 (5)
O3—N2	1.239 (4)	C5—H5	0.9500
O4—N2	1.222 (4)	C6—H6	0.9500
N1—C7	1.383 (5)	C7—C8	1.492 (5)
N1—C1	1.401 (5)	C8—H8A	0.9800
N1—H1N	0.87 (5)	C8—H8B	0.9800
N2—C2	1.467 (4)	C8—H8C	0.9800
C1—C6	1.400 (5)	C9—H9A	0.9800
C1—C2	1.405 (5)	C9—H9B	0.9800
C2—C3	1.404 (5)	C9—H9C	0.9800
C3—C4	1.372 (5)		
			
C4—O2—C9	117.1 (3)	C6—C5—H5	119.5
C7—N1—C1	127.4 (3)	C4—C5—H5	119.5
C7—N1—H1N	118 (3)	C5—C6—C1	121.7 (3)
C1—N1—H1N	113 (3)	C5—C6—H6	119.1
O4—N2—O3	122.6 (3)	C1—C6—H6	119.1
O4—N2—C2	117.9 (3)	O1—C7—N1	123.6 (3)
O3—N2—C2	119.6 (3)	O1—C7—C8	123.8 (3)
C6—C1—N1	121.6 (3)	N1—C7—C8	112.7 (3)
C6—C1—C2	116.2 (3)	C7—C8—H8A	109.5
N1—C1—C2	122.2 (3)	C7—C8—H8B	109.5
C3—C2—C1	122.4 (3)	H8A—C8—H8B	109.5
C3—C2—N2	115.6 (3)	C7—C8—H8C	109.5
C1—C2—N2	122.0 (3)	H8A—C8—H8C	109.5
C4—C3—C2	119.2 (3)	H8B—C8—H8C	109.5
C4—C3—H3A	120.4	O2—C9—H9A	109.5
C2—C3—H3A	120.4	O2—C9—H9B	109.5
O2—C4—C3	124.9 (3)	H9A—C9—H9B	109.5
O2—C4—C5	115.7 (3)	O2—C9—H9C	109.5
C3—C4—C5	119.4 (3)	H9A—C9—H9C	109.5
C6—C5—C4	121.0 (3)	H9B—C9—H9C	109.5
			
C7—N1—C1—C6	25.4 (5)	C9—O2—C4—C3	6.1 (5)
C7—N1—C1—C2	−154.6 (3)	C9—O2—C4—C5	−174.3 (3)
C6—C1—C2—C3	−1.8 (5)	C2—C3—C4—O2	179.1 (3)
N1—C1—C2—C3	178.2 (3)	C2—C3—C4—C5	−0.4 (5)
C6—C1—C2—N2	180.0 (3)	O2—C4—C5—C6	−179.9 (3)
N1—C1—C2—N2	−0.1 (5)	C3—C4—C5—C6	−0.3 (5)
O4—N2—C2—C3	−10.9 (4)	C4—C5—C6—C1	−0.1 (5)
O3—N2—C2—C3	168.8 (3)	N1—C1—C6—C5	−178.9 (3)
O4—N2—C2—C1	167.5 (3)	C2—C1—C6—C5	1.1 (5)
O3—N2—C2—C1	−12.8 (5)	C1—N1—C7—O1	−3.9 (6)
C1—C2—C3—C4	1.5 (5)	C1—N1—C7—C8	175.8 (3)
N2—C2—C3—C4	179.9 (3)		

### Hydrogen-bond geometry (Å, º)

**Table:**

*D*—H···*A*	*D*—H	H···*A*	*D*···*A*	*D*—H···*A*
N1—H1*N*···O3	0.87 (5)	1.92 (5)	2.632 (4)	137 (4)
C5—H5···O2^i^	0.95	2.48	3.418 (4)	171
C6—H6···O1	0.95	2.30	2.864 (4)	117
C8—H8*B*···O3^ii^	0.98	2.64	3.578 (4)	160
C8—H8*C*···O4^iii^	0.98	2.63	3.546 (4)	156

Symmetry codes: (i) −*x*+1, −*y*, −*z*+1; (ii) −*x*+1/2, *y*+1/2, −*z*+3/2; (iii) *x*+1/2, −*y*+5/2, *z*+1/2.
